# Intensive care inequity in Rio de Janeiro: the effect of spatial distribution of health services on severe acute respiratory infection

**DOI:** 10.5935/0103-507X.20200012

**Published:** 2020

**Authors:** Sandro Javier Bedoya-Pacheco, Romeu Ferreira Emygdio, José Antônio Sena do Nascimento, Jorge André Marques Bravo, Fernando Augusto Bozza

**Affiliations:** 1 Department of Epidemiology and Quantitative Methods in Health, Escola Nacional de Saúde Pública Sérgio Arouca, Fundação Oswaldo Cruz - Rio de Janeiro (RJ), Brazil.; 2 Coordination of Natural Resources and Environmental Studies, Instituto Brasileiro de Geografia e Estatística - Rio de Janeiro (RJ) – Brazil.; 3 Geosciences Board, Instituto Brasileiro de Geografia e Estatística - Rio de Janeiro (RJ), Brazil.; 4 Instituto Nacional de Câncer José Alencar Gomes da Silva - Rio de Janeiro (RJ), Brazil.; 5 Instituto Nacional de Infectologia Evandro Chagas, Fundação Oswaldo Cruz - Rio de Janeiro (RJ), Brazil.

**Keywords:** Intensive care units/organization & administration, Quality of health care, Respiratory tract infections, Health status disparities, Unidades de terapia intensiva/organização & administração, Qualidade da assistência à saúde, Infecções respiratórias, Disparidades nos níveis de saúde

## Abstract

**Objective:**

To analyze the distribution of adult intensive care units according to geographic region and health sector in Rio de Janeiro and to investigate severe acute respiratory infection mortality in the public sector and its association with critical care capacity in the public sector.

**Methods:**

We evaluated the variation in intensive care availability and severe acute respiratory infection mortality in the public sector across different areas of the city in 2014. We utilized databases from the National Registry of Health Establishments, the Brazilian Institute of Geography and Statistics, the National Mortality Information System and the Hospital Admission Information System.

**Results:**

There is a wide range of intensive care unit beds per capita (from 4.0 intensive care unit beds per 100,000 people in public hospitals in the West Zone to 133.6 intensive care unit beds per 100,000 people in private hospitals in the Center Zone) in the city of Rio de Janeiro. The private sector accounts for almost 75% of the intensive care unit bed supply. The more developed areas of the city concentrate most of the intensive care unit services. Map-based spatial analysis shows a lack of intensive care unit beds in vast territorial extensions in the less developed regions of the city. There is an inverse correlation (r = -0.829; 95%CI -0.946 to -0.675) between public intensive care unit beds per capita in different health planning areas of the city and severe acute respiratory infection mortality in public hospitals.

**Conclusion:**

Our results show a disproportionate intensive care unit bed provision across the city of Rio de Janeiro and the need for a rational distribution of intensive care.

## Abstract (pt)

**Objetivo:**

Analisar a distribuição das unidades de terapia intensiva para adultos, segundo a região geográfica e o setor sanitário no Rio de Janeiro, e investigar a mortalidade por infecção respiratória aguda grave no setor público e sua associação com a capacidade de terapia intensiva no setor público.

**Métodos:**

Avaliamos a variação da disponibilidade de terapia intensiva e a mortalidade por infecção respiratória aguda grave no setor público em diferentes áreas da cidade em 2014. Utilizamos as bases de dados do Cadastro Nacional de Estabelecimentos de Saúde, do Instituto Brasileiro de Geografia e Estatística, do Sistema de Informações sobre Mortalidade e do Sistema de Informações Hospitalares do SUS.

**Resultados:**

Foi ampla a variação na disponibilidade de leitos em unidades de terapia intensiva *per capita* (desde 4,0 leitos de terapia intensiva por 100 mil habitantes em hospitais públicos na zona oeste até 133,6 leitos em unidades de terapia intensiva por 100 mil habitantes nos hospitais privados na zona central) na cidade do Rio de Janeiro. O setor privado respondeu pelo suprimento de quase 75% dos leitos em unidades de terapia intensiva. Uma análise espacial com base em mapas mostrou falta de leitos em unidades de terapia intensiva em vastas extensões territoriais nas regiões menos desenvolvidas da cidade. Houve correlação inversa (r = -0,829; IC95% -0,946 - -0,675) entre a quantidade de leitos públicos em unidade de terapia intensiva *per capita* em diferentes áreas de planejamento em saúde na cidade e a mortalidade por infecção respiratória aguda grave em hospitais públicos.

**Conclusão:**

Nossos resultados mostram disponibilidade desproporcional de leitos em unidades de terapia intensiva na cidade do Rio de Janeiro e a necessidade de uma distribuição racional da terapia intensiva.

## INTRODUCTION

The health sector in Brazil has undergone profound changes over the last decades. Universal vaccination and prenatal care have been virtually achieved in recent decades. Nevertheless, the efficiency of the system and the quality of care among different social strata are still a challenge.^(1)^In the past few years, the country has invested in public policies to mitigate these inequalities.^(2,3)^ However, these strategies are often insufficient due to the growing demand for health services and are constantly threatened by economic instability.^(4,5)^These policies have been focused on a primary health care model.^(2)^ In contrast, disparities remain substantial in tertiary care, in which expensive procedures including complex surgeries and hospitalizations in intensive care units (ICU), are performed.^(6,7)^Several studies show how social inequalities impact the prevalence and distribution of health risk factors.^(8-10)^Socioeconomic conditions shape the population health profile, lifestyle and behavior^(11-15)^ and have a significant impact on the morbidity and premature mortality of the disadvantaged,^(16-19)^ placing an extra burden over the health care system. Although rich countries have socioeconomic inequalities with health repercussions,^(20,21)^ there is a substantially larger gap in Brazil due to the larger proportion of the population living in unfavorable economic conditions.^(22)^

Rio de Janeiro is one of the largest cities in South America, with a population close to 6.5 million people. The city is geographically divided into four zones: South, Center, North, and West. Among the four zones, the South Zone has better socioeconomic indicators. All four zones display, to a greater or lesser extent, disparities in income distribution among segments of their populations.^(23)^ Informal settlements (shanty towns) can be observed throughout the territory of the city.^(24)^ Primary care coverage increased from 3.5% at the end of the past century to 40% in 2013.^(25)^ Tertiary care, however, has failed to keep up with the expansion of primary care in the city.

There is a global variation in the capacity to provide critical care, with substantial discrepancies even among developed countries.^(26)^Because critical care services represent one of the most expensive components of tertiary care, they must be distributed in a rational manner to maintain an adequate balance, avoiding discrepancies with the potential of wasting precious resources (in case of oversupply) or contributing to an increase in the burden of disease (in case of shortage).^(27)^

Considering these public health issues and bearing in mind future proposals, we conducted the present study, aiming to analyze the spatial distribution of adult ICU beds according to geographic regions in Rio de Janeiro and health sectors (public or private), using indicators and maps adjusted by population size. Finally, we analyzed severe acute respiratory infection (SARI) mortality in the public sector and its association with the public sector critical care capacity.

## METHODS

This study was approved by the Research Ethics Committee of the *Instituto Nacional de Infectologia Evandro Chagas* (INI) of the *Fundação Oswaldo Cruz* (FIOCRUZ) under the number 30865214.6.0000.5262. The ICUs and respective institutions studied did not have their identification revealed during the study.

We utilized the databases of the National Registry of Health Institutions (*Cadastro Nacional dos Estabelecimentos de Saúde* - CNES), which is available at http://cnes.datasus.gov.br/, accessed July 2015 for the identification of all adult ICU beds in general hospitals existing in the city of Rio de Janeiro in 2014.

### Intensive care unit definition

We used the minimum standards required by the national health surveillance agency (Anvisa - *Agência Nacional de Vigilância Sanitária*) for the definition of an ICU: (i) the existence of continuous monitoring and the presence of life support devices and (ii) permanent nursing and medical specialists.^(28)^As this research aimed to evaluate the capacity of critical care for severe acute respiratory diseases, we excluded pediatric and neonatal ICU beds as well as ICU beds located in specialty hospitals, including oncology, chronic care, burn, cardiology, orthopedics, and plastic surgery units. We also excluded intensive care beds located in hospitals with restricted access to a specific population, namely, military and penitentiary hospitals. Thus, we analyzed only general hospitals intended for acute care in the public and private sectors of the city of Rio de Janeiro.

### Disaggregation of intensive care unit by size and administrative area

After identifying hospitals, we classified them according to their general administrative domain (i.e., private or public) and specific administrative domain (i.e., federal, state or city hospital). We also classified hospitals according to size. For that purpose, we used the definition from the Brazilian Ministry of Health: large hospitals are those with 151 to 500 beds. Medium sized hospitals are hospitals with 51 to 150 beds, and small hospitals are those with up to 50 beds.^(29)^

### Population indicators for intensive care unit beds

Population projections were made in partnership with the *Instituto Brasileiro de Geografia e Estatística* (IBGE). Different populations were grouped according to city districts, geographic regions and health planning areas (PA), according to the definition of the Rio de Janeiro Department of Health.

For the construction of population indicators, we used the bed coefficient per 100,000 people: a) Number of adult ICU/resident population in the neighborhood aged over 17 years x 100,000 (adult population). We adopted this formula for each unit of analysis (e.g., administrative domain, region, hospital size, etc.).

### Spatial distribution and map construction

We used the cartographic database from the IBGE for the construction of maps (available at http://mapas.ibge.gov.br/pt/bases-e-referenciales/bases-cartograficas/malhas-digitais, accessed in July 2015).

The shapefile file from the State of Rio de Janeiro was manipulated in ArcGIS version 10.2.2 (ERSI, Redlands, CA) and Terraview version 4.2.2 (São José dos Campos, SP: INPE) to create thematic maps that indicated the coefficient of ICU beds by district in the city of Rio de Janeiro and Voronoi diagrams for public and private units.

Voronoi diagrams were used as instruments for the definition of coverage areas of hospital units.^(30)^ Given a number of points on a given plane, a Voronoi diagram divides that plane according to the “nearest neighbor rule”; that is, each point is associated with the region of the plane closest to that point. The edges of the polygons constructed through the points are equidistant from their respective generating points.^(31)^The area of ​​coverage of a point (a centroid), that is, of a hospital unit, can thus be estimated through Voronoi diagrams. For the generation of centroids, each discriminated acute care hospital was georeferenced through the CNES addresses and Google Earth (Google Inc., Mountain View, CA). The geographic coordinates of the centroid for each hospital unit were exported to the ArcGIS program version 10.2.2 (ERSI, Redlands, CA) for the generation of Voronoi diagrams.

### Statistical analysis of the association of critical care capacity with hospital severe acute respiratory infection mortality in different health planning areas

The city health department further subdivides Rio de Janeiro into ten PAs. Health planning area 1 concentrates the largest public health apparatus installed in the city. Health planning area 2.1 has the highest human development index in the city. Health planning area 2.2 has a profile very close to that found in PA 2.1. The PAs 3.1, 3.2 and 3.3 together are the most populous areas of the city; half of the city’s informal settlements are in this region (37.9%). Health planning area 4 is the second largest area with 294km^2^. Health planning areas 5.1, 5.2 and 5.3 are the second most populous areas of the city and constitute a recent vector of urban expansion for low- and middle-income populations.^(32)^

We collected all hospital admission permits (with an admission diagnosis of acute upper respiratory infections, influenza and pneumonia, other acute lower respiratory infections, other upper respiratory diseases, necrotic and suppurative diseases of the lower airways in persons over 17 years old) issued by public hospitals in each PA. The hospital admission permits database in Rio de Janeiro was exclusively available for public hospitals (available at http://tabnet.rio.rj.gov.br/) for the year 2014). There is no comparable database for private hospitals in Rio de Janeiro. Therefore, mortality in the private sector could not be evaluated in this study.

We analyzed the proportion of hospitalization deaths in public hospitals by these groups of diseases, according to the different PAs of the city, thus generating different hospital mortality rates for SARI. The availability of adult ICU beds *per capita* in each PA was correlated with hospital mortality for SARI using the Pearson correlation coefficient. All data management and statistical analyses were performed using Excel 2013 (Microsoft, Redmond, WA) and IBM Statistical Package for Social Science (SPSS) for Windows, version 21.0 (Armonk, NY: IBM). The tests were performed at the 5% level of significance. We undertook the same analysis described above for SARI in another critical condition (ischemic heart disease) as a comparison parameter to assess the robustness of our assessment.

We utilized PAs as a regression analysis unit for greater information disaggregation. Health planning areas correspond to the smaller territorial (demographic) regions with information that allowed the correlation between the number of ICU beds and SARI mortality. The obtained data are presented in cross tables and scatter plots.

## RESULTS

### Ratios, proportions and coefficients

The city of Rio de Janeiro in 2014 had 13,013 adult hospitalization beds distributed in 87 health facilities, with a rate of 264 hospitalization beds per 100,000 people, as the population was estimated as 4,917,518 inhabitants. The number of active ICU beds available for the adult population was 1,896 (14.6%), with a ratio of 1 ICU bed to every 5.9 hospital beds. The distribution of ICU beds and the ratio of ICU beds to general beds, according to region, administrative domain (public or private sector) and unit size, are presented in table 1.

**Table 1:** Intensive care unit bed distribution according to conventional hospital beds in acute care hospitals in the city of Rio de Janeiro in 2014

Variable	Hospitals	Conventional beds	ICU beds	Ratio*
Total	87	13,013	1,896	1:5.9
Region (Zone)				
South	16	1,884	351	1:4.4
Center	12	2,241	313	1:6.2
North	37	5,819	773	1:6.5
West	22	3,069	459	1:5.7
Health sector				
Public	23	6,175	503	1:11.3
Federal	8	2,489	171	1:13.6
State	7	1,791	167	1:9.7
City	8	1,895	165	1:10.5
Private†	64	6,838	1,393	1:3.9
Hospital size‡				
Large	28	8,240	839	1:8.8
Medium	40	4,158	824	1:4
Small	19	615	233	1:1.6

ICU - intensive care unit.

* Intensive care unit beds/conventional beds ratio.

† 15 beds in two private hospitals are leased to the public sector.

‡ Hospital size: large (> 151 beds), medium (51 - 150 beds), small (< 50 beds).

Results expressed as n.

The highest concentrations of conventional and ICU beds are located in private (74%) and medium/large hospitals. Most private hospitals with ICU beds are of medium and small sizes, whereas public hospitals are mostly large ones. In all regions of the city, most ICU beds belong to the private sector (65% to 82%). The ratio of ICU beds to conventional beds is higher in the private sector. The same trend is observed in all regions of the city (Table 2).

**Table 2:** Distribution of intensive care unit beds according to region and unit size in private and public sector in the city of Rio de Janeiro in 2014

Variable	Total ICU beds	Private sector	Public sector	Ratio*
Total	1,896	1,393 (73.5)	503 (26.5)	1:2.8
Region (Zone)				
South	351	273 (77.8)	78 (22.2)	1:3.5
Center	313	241 (77.0)	72 (23.0)	1:3.3
North	773	499 (64.6)	274 (35.4)	1:1.8
West	459	380 (82.8)	79 (17.2)	1:4.8
Hospital size†				
Small	233	197 (84.5)	36 (15.5)	1:5.4
Medium	824	739 (89.7)	85 (10.3)	1:8.6
Large	839	457 (54.5)	382 (45.5)	1:1.2

ICU - intensive care unit.

* Public/Private intensive care unit bed ratio.

† Hospital size: large (> 151 beds), medium (51 - 150 beds), small (< 50 beds).

Results expressed as n or n (%).

The number of adult intensive care beds (public and private) in the city of Rio de Janeiro is 38.6 beds per 100,000 people (Table 3). The South Zone (the region of the city with the highest purchasing power) displays a coefficient of ICU beds per capita of 63.9/100,000 people, higher than the West and North Zones (23.3/100,000 people and 35.7/100,000 people, respectively), with lower purchasing power. The proportion of ICU beds available for the public sector is much smaller.

**Table 3:** Adult conventional and intensive care beds coefficients per capita (acute care hospitals) in different regions in the city of Rio de Janeiro in 2014

Total of beds					Beds per 100,000 people		
Region (zone)	Conventional	ICU*	ICU (public)	Population (over 17 years)	Total	ICU*	ICU (public)
South	1,842	351	78	549,500	335.2	63.9	14.2
Center	2,283	313	72	234,330	974.3	133.6	30.7
North	5,936	773	274	2,167,797	273.8	35.7	12.6
West	2,952	459	79	1,965,891	150.2	23.3	4.0
Total	13,013	1896	503	4,917,518	264.6	38.6	10.2

ICU - intensive care unit.

* Includes beds in the private and public sectors.

Results expressed as n.

### Spatial distribution and Voronoi diagrams

**Figure 1:**
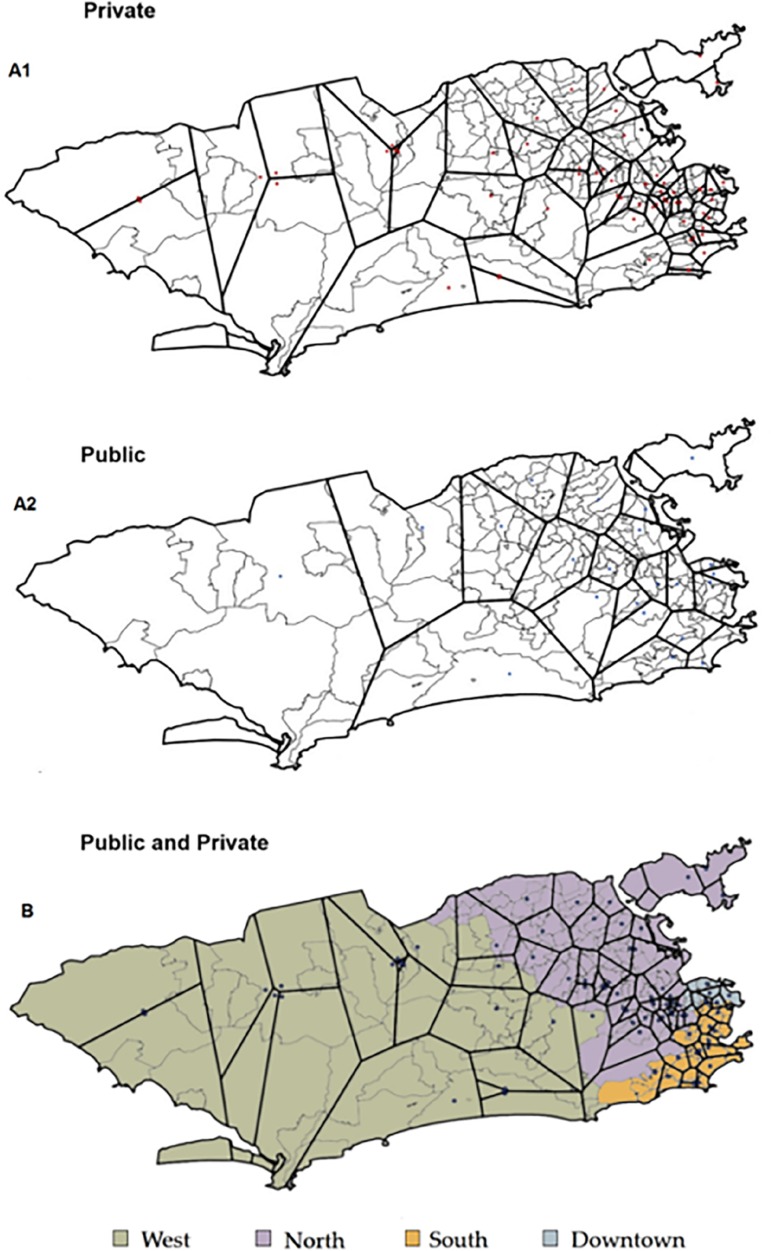
City districts, zones and Voronoi polygons. A1 - private sector; A2 - public sector; B - private and public sectors. Thick lines = Voronoi polygons. Thin lines = city districts. Dots = centroid.

The Voronoi diagrams (Figure 1) show the inequalities in the distribution of ICU beds in different districts of the city. Large polygons represent areas with low coverage. Smaller polygons represent areas with better coverage. Most of the city districts show low ICU bed coverage, in either public or private sector. The analysis of catchment areas of coverage by Voronoi diagrams shows that the West and North Zones are the critical regions that present most districts with low ICU coverage, whereas the South Zone displays the highest number of polygons that are smaller in size, demonstrating better coverage for both the private and public sectors.

### Public sector intensive care unit beds and severe acute respiratory infection mortality in different health planning areas

The geographic zones (Center, South, North, and West) are a convenient way to evaluate the city in the traditional historical division. However, each zone is too broad and covers a wide area, aggregating profoundly different parts of the city in socioeconomic terms. Therefore, the use of smaller areas, such as the PA, increases the sensitivity of the analysis.

**Figure 2:**
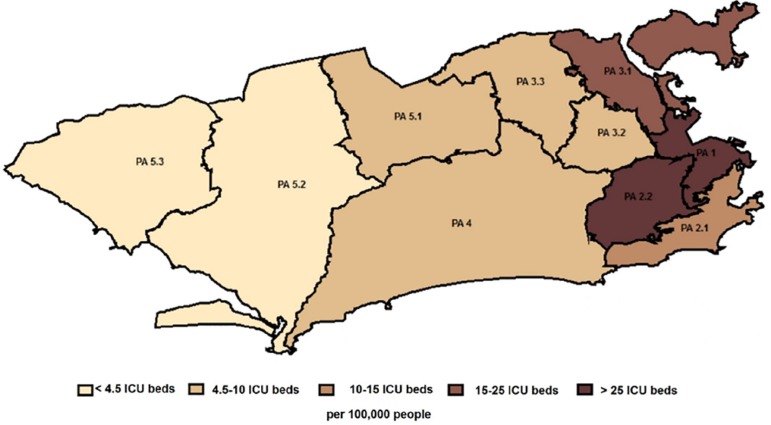
Health planning areas of the city and intensive care unit beds per capita in different planning areas. PA - health planning area; ICU - intensive care unit.

**Figure 3:**
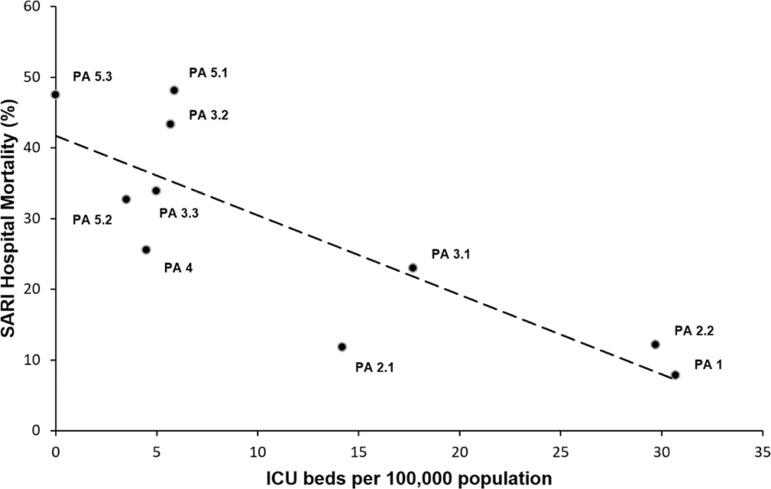
Correlation between intensive care unit beds per capita and public hospital severe acute respiratory infection mortality in different health planning areas. SARI - severe acute respiratory infection; PA - health planning area; ICU - intensive care unit.

Table 4 shows the number of hospital admissions due to respiratory diseases in adults in public hospitals, the hospital deaths due to these causes and the public sector coefficient of adult intensive care in different health PAs of the city of Rio de Janeiro in 2014. The admission database exists exclusively in the public sector. Thus, we cannot estimate private hospital mortality with the current study design. There is ample variation in ICU beds per capita in different health PAs of the city (Figure 2). A strong inverse correlation (r = -0.829; 95% confidence interval - 95%CI -0.946 to -0.675) can be observed between ICU beds per capita and hospital mortality due to acute infectious respiratory disease in the different PAs (Figure 3). It is evident that, except for the first four PAs, all the others have critical care bed rates below 6.0 beds/100,000 people. Similarly, we found an inverse correlation (r = - 0.635; 95%CI -0.860 to -0.003) between ICU beds per capita and hospital mortality due to ischemic heart disease.

**Table 4:** Adult infectious respiratory disease admissions in public hospitals, public hospital mortality for respiratory infectious diseases and adult public intensive care unit beds per capita in different health planning areas in the city of Rio de Janeiro in 2014

PA	Admissions*	Deaths*	Hospital mortality	Intensive care beds per 100,000 people
PA 1	370	29	7.8	30.7
PA 2.1	534	63	11.8	14.2
PA 2.2	767	93	12.1	29.7
PA 3.1	1,231	283	23.0	17.7
PA 3.2	307	133	43.3	5.7
PA 3.3	971	329	33.9	5.0
PA 4	376	96	25.5	4.5
PA 5.1	416	200	48.1	5.9
PA 5.2	101	33	32.7	3.5
PA 5.3	280	133	47.5	0.0
Total	5,353	1,392	26.0	10.2

ICU - intensive care unit; PA - health planning area.

* Acute upper respiratory tract infections, influenza and pneumonia, other acute lower respiratory tract infections, other diseases of the upper airways, necrotic and suppurative diseases of the lower airways.

Results expressed as n or %.

## DISCUSSION

Our results show an uneven ICU bed distribution across the city of Rio de Janeiro, with lower coverage in the most underprivileged areas of the city. Other authors have utilized geographic information systems to identify a bed shortage in the West Zone of the city.^(33)^ We found this distribution straightforwardly displayed on Voronoi diagrams. The private sector accounts for almost 75% of the ICU bed supply in the city. Additionally, we found an inverse correlation (r = -0.829; 95%CI -0.946 to -0.675) between public ICU beds per capita in different PAs of the city and public hospital SARI mortality.

Inequity in ICU bed distribution can be regarded as an indicator of inequality of access, hampering the ability of the system to solve various illnesses, and may have a substantial impact on early mortality. Significant geographical variances in health services may have the effect of worsening the health conditions of the underlying populations. Studies show that disparities in service delivery have an impact on the burden of disease and mortality, especially in economically disadvantaged groups, with an inverse correlation between health and inequality. Studies have shown higher infant and general mortality rates, as well as lower life expectancies, in areas with a higher concentration of poor populations.^(15,17,18,34)^

There are discrepancies in the literature regarding the ideal number of ICU beds for different populations. There is ample variation among countries and even among regions of the same country. The number of ICU beds will vary according to factors such as the burden of disease or the type of coverage in the system.^(26,35-38)^ Even in developed countries, there is an eight-fold difference in the availability of ICU beds, ranging from 3 to 25 beds per 100,000 people.^(39)^ In Europe, Paris has 8.38 ICU beds per 100,000 people. In North America, Boston displays 18.85 ICU beds per 100,000 people. Liaocheng, China has 2.8 ICU beds per 100,000 people.^(40)^In our study, we found 38.6 ICU beds per 100,000 people in Rio de Janeiro; a high critical care capacity in comparison to many cities in the developed world.

We found a trend towards the creation of private institutions and the expansion of ICU beds within those establishments. Although the proportion of conventional beds is similar in the public and private sectors, ICU beds are present in a larger proportion in the private sector (74%), with an ICU bed/conventional bed ratio three times higher in the private sector (20 in the private sector and 8 in the public sector). Nearly three-quarters of the critical care capacity for adult acute care in Rio de Janeiro is allocated to the private sector. Other authors have described inequality in the neonatal ICU bed distribution in the state, with bed shortages in the public sector, excesses in the private sector, and a large concentration in the metropolitan area of the state.^(41)^ Similar findings were also observed in other middle-income countries, including South Africa, where 75% of intensive care beds are available in the private system, although the intensive care therapy coefficients of 2.4 and 7.2 ICU beds/100,000 people for the public and private sectors, respectively, are significantly lower than those observed in Brazil.^(42)^

It has been indicated that hospitalizations in Brazil are associated with private health insurance coverage, characterizing overuse of private insurance. This trend is in marked contrast to reduced access to procedures that require hospitalization among the poorest segments of society that do not carry this type of coverage.^(43)^ We believe that a similar tendency occurs in the ICU admission system. The toll to the system becomes even more unambiguous given that a portion of ICU patients might not need this kind of care. Private sector fees on individuals can compromise a significant part of society savings as much as government debts to the private sector (which offer part of their ICU beds to the public sector at expensive rates) can deteriorate public finances.

We found a strong inverse correlation between ICU bed per capita and respiratory disease mortality in the public sector. Additionally, as a comparison measure, we analyzed the mortality rate in the different PAs for another critical illness (ischemic heart disease). We observed a similar trend for this condition in the public sector. However, correlation does not necessarily imply causation. Our findings do not suggest that decreased ICU coverage is responsible for a higher mortality in different PAs in the public sector (this development may be due to other variables, such as the burden of disease in different populations); they merely show the necessity of further investigation on this matter.

Another limitation of this study is specifically related to the fact that each district, PA, or region does not constitute a homogeneous socioeconomic area, mainly due to the presence of informal settlements.

We assume that the resident populations use the closest services, which may not correspond to reality. This may also influence ICU bed coverage rates, which may not match the services used by a population in a given region. We do not have data on the residence of individuals who died in hospitals. The complexity of the access to health in Rio de Janeiro and its peculiar geography did not allow us to establish diagrams other than Voronoi diagrams. However, in a city of six and a half million inhabitants and 1,200km^2^, we assume a tendency of the population to look for the closest services. Thus, the inability to analyze the flow of patients across different city areas is another weakness in our study. In our analysis, we did not consider the diversity within the areas studied or the real access of the population to health services. One author pointed out an important flow of West Zone infants to the Center Zone.^(44)^ Nevertheless, there is little doubt that a geographical inequity in ICU bed supply is a clear phenomenon and that more specific studies are needed, especially those designed to demonstrate the health effects of these inequalities.

The lack of data on deaths in private hospitals is another limitation that weakens our results. Admission permit forms are mandatory in the public sector. These forms hold information regarding disease prevalence, mortality and other indicators that, in turn, feed a nationwide database. The lack of comparable data in the private sector makes any comparisons between the systems difficult to achieve. A mandatory comparable database in the private sector would allow such analyses, contributing to the rational planning of the health system.

Demographic and epidemiological shifts as well as increased population longevity will change the morbidity and mortality profile and will encompass the need for reinforcing tertiary care. We must be careful not to incorporate a veiled form of discrimination that asserts that the underprivileged population needs only basic health care.

In addition to the need for expanding universal coverage for socially and economically disadvantaged sectors of society, a new proposal for the organization of critical care in Rio de Janeiro is required. Planning hierarchical reference regions may simplify the access of patients requiring critical care. However, the empirical basis for the implementation of such reference regions is lacking. Thus, we need data regarding the flow of patients, the significant geographical borders and the observation of the effects of the critical care capacity considering the population burden of disease.

## CONCLUSION

Our study demonstrates the distributions of critical care beds in the city of Rio de Janeiro, with substantial variability in the critical care capacity among different areas of the city. There is low coverage of intensive care unit beds in more disadvantaged areas of the city. These results highlight the need to increase the awareness of public health managers to coherently adjust the supply of tertiary care resources.
